# 3Es for AI: Economics, Explanation, Epistemology

**DOI:** 10.3389/frai.2022.833238

**Published:** 2022-03-29

**Authors:** Nitasha Kaul

**Affiliations:** Centre for the Study of Democracy (CSD), University of Westminster, London, United Kingdom

**Keywords:** artificial intelligence (AI), explainable artificial intelligence (XAI), theories of explanation, epistemology, philosophy, economics, transdisciplinary, sociotechnical knowledges

## Abstract

This article locates its roots/routes in multiple disciplinary formations and it seeks to advance critical thinking about an aspect of our contemporary socio-technical challenges by bracketing three knowledge formations—artificial intelligence (AI), economics, and epistemology—that have not often been considered together. In doing so, it responds to the growing calls for the necessity of further transdisciplinary engagements that have emanated from work in AI and also from other disciplines. The structure of the argument here is as follows. First, I begin by demonstrating how and why explanation is a problem in AI (“XAI problem”) and what directions are being taken by recent research that draws upon social sciences to address this, noting how there is a conspicuous lack of reference in this literature to economics. Second, I identify and analyze a problem of explanation that has long plagued economics too as a discipline. I show how only a few economists have ever attempted to grapple with this problem and provide their perspectives. Third, I provide an original genealogy of explanation in economics, demonstrating the changing nature of what was meant by an explanation. These systematic changes in consensual understanding of what occurs when something is said to have been “explained”, have reflected the methodological compromises that were rendered necessary to serve different epistemological tensions over time. Lastly, I identify the various relevant historical and conceptual overlaps between economics and AI. I conclude by suggesting that we must pay greater attention to the epistemologies underpinning socio-technical knowledges about the human. The problem of explanation in AI, like the problem of explanation in economics, is perhaps not only, or really, a problem of satisfactory explanation provision alone, but interwoven with questions of competing epistemological and ethical choices and related to the ways in which we choose sociotechnical arrangements and offer consent to be governed by them.

“The most secure part of our present knowledge is knowing what it is that we cannot know or do” [Louis Fein, *Impotence Principles for Machine Intelligence*, 1968,p. 443, in Mendon-Plasek (2021), p. 53]

“No science can be more secure than the unconscious metaphysics which it tacitly presupposes” [Alfred North Whitehead, *Adventures of Ideas*, 1933, p. 197]

“Economists were present at the creation of the cyborg sciences, and, as one would expect, the cyborg sciences have returned the favor by serving in turn to remake the economic orthodoxy in their own image” [Philip Mirowski, *Machine Dreams: Economics Becomes a Cyborg Science*, 2002, p. 6]

## Introduction

This article locates its roots/routes in multiple disciplinary formations and it seeks to advance critical thinking about an aspect of our contemporary socio-technical challenges by bracketing three knowledge formations—artificial intelligence (AI), economics, and epistemology—that have not often been considered together. In doing so, it responds to the multiple calls for the necessity of further transdisciplinary engagements that have emanated from work in AI (for instance, Arrieta et al., 2019, p. 85, 102; Miller, 2019, p. 34; Paez, 2019, p. 457), especially as they relate to the ongoing search for XAI (explainable artificial intelligence) or the problems of AI bias. Similar concerns have now begun to resonate in economics (see Ludwig and Mullainathan, 2021, p. 87), and there is a need for transdisciplinary approaches in order to excavate the epistemological foundations as well as the cultural and scientific implications of AI (see Mariotti, 2021, p. 566–68).

The structure of the argument here is as follows. First, I begin by demonstrating how and why explanation is a problem in AI (“XAI problem”) and what directions are being taken by recent research that draws upon social sciences to address this, noting how there is a conspicuous lack of reference in this literature to economics. Second, I identify and analyze a problem of explanation that has long plagued economics too as a discipline. I show how only a few economists have ever attempted to grapple with this problem and provide their perspectives. Third, I provide an original genealogy of explanation in economics, demonstrating the changing nature of what was meant by an explanation. These systematic changes in consensual understanding of what occurs when something is said to have been “explained”, have reflected the methodological compromises that were rendered necessary to serve different epistemological tensions over time. Lastly, I identify the various relevant historical and conceptual overlaps between economics and AI. I conclude by suggesting that we must pay greater attention to the epistemologies underpinning socio-technical knowledges about the human. The problem of explanation in AI, like the problem of explanation in economics, is perhaps not only, or really, a problem of satisfactory explanation provision alone, but interwoven with questions of competing epistemological and ethical choices and related to the ways in which we choose sociotechnical arrangements and offer consent to be governed by them.

## AI, XAI, and Social Sciences

AI is ubiquitous. From scholarly work to human experience to media reports, the background, applications, and concerns about AI are everywhere (De Angelis, 2014; Darlington, 2017; Doshi-Velez et al., 2017; Chipman, 2018; Gunning and Aha, 2019; Rai, 2020; Tyagi, 2020; Verma et al., 2020; Brown, 2021)^1^. AI applications are a part of decision-making in healthcare, transportation, logistics, marketing, social media, recruitment, entertainment, law enforcement, finance, military, policing, security, education, communication and more. Yet, the many complex and important problems solved by 'deep learning' methods are not necessarily appropriate for other domains where the inscrutable techniques create access barriers to transparency and accountability (Knight, 2017). Hence, today, we face the problem of “black-box ontology” in AI, otherwise known as the “problem of explanation” in AI. The machine learning techniques responsible for advances in AI are not explainable even by experts and there is a gap between research and practice (Arya et al., 2019). Rodu and Baiocchi (2021, p. 1) write, “some of the most successful algorithms are so complex that no person can describe the mathematical features of the algorithm that gives rise to the high performance (a.k.a. ‘black box algorithms')”. AI researchers and others confront situations where machines that may produce decision outcomes that may be useful, but we may not be able to access how or why these were reached (on this issue of relative merits of careful theoretical justification vs. rapid performance improvement, see Rodu and Baiocchi, 2021, p. 17–18).

Aside from rule-based systems, present-day AI systems are composed of unsupervised machine learning and non-symbolic deep neural networks that learn from massive amounts of data in order to make predictions. The network's reasoning is based on complex and layered calculations that are inaccessible to humans, so that their own designers, let alone the end users, are unable to have an explanation for such automated decision-making. Enter, need for XAI, or explainable artificial intelligence. Explainability may not be vital in all domains, but its importance becomes underlined in domains with high stakes such as AI applications in medical diagnostics, autonomous vehicles, finance, or defense. This is an evolving literature without a clear consensus; for instance, see Durán and Jongsma (2021) for an evaluation of the challenging but nonetheless useful role of black box algorithms in medical AI.

In the last few years, the Defense Advanced Research Projects Agency (DARPA) at Department of Defense, U.S focused its XAI program on ways for AI systems to provide explanations. The DARPA XAI program refers to the success in machine learning leading to AI applications and the promise of autonomous systems that “perceive, learn, decide, and act on their own”, but that are currently limited by their “inability to explain their decisions and actions to human users”, thus the need for explainable AI, and especially explainable machine learning (Turek, n.d.).

AI scholarship has two distinct ways in which explanation is considered: one, work that draws upon the idea of explanation in other disciplines as relevant for generating XAI and two, work that pulls together the various ways that explanation has been understood in XAI. What emerges is that within AI, explanation is often seen in specifically functional ways, primarily as a route to increasing the acceptability of machine learning outcomes in order to increase the trust in decision-making by machines so as to help the uptake of AI across sectors and economies. A specific reason for the focus on explanation has also been the policy environment and the advance of legislation such as the European Union General Data Protection Regulation (GDPR) where several articles (for instance, articles 13, 14, 15, 22) provide recourse/remedy/safeguards to individuals affected by entirely automated decision-making (The Royal Society, 2019, p. 27–30, see also The Alan Turing Institute, 2021). Often referred to as “the right to explanation”, the exact conditions under which it is legally binding are unclear (see Wachter et al., 2017), but it does bring to the surface the questions of accountability in law when solely automated decision-making has an adverse, disproportionate, or discriminatory effect on specific individuals.

Referring to a demand for transparency in algorithmic decision-making, Rauber et al. (2019, p. 10–11) state, “we should consider the main question in the field to be: what is an explanation?” The idea of what is meant by an explanation is, of course, far from straightforward and varies across disciplines. DARPA's own literature review on explanation by Mueller et al. (2019) provides an encyclopedic overview of references to work on explanation as understood across a variety of disciplines (but not economics), drawing out links considered relevant for XAI. They provide a synopsis of key XAI concepts, kinds of AI systems (rule-based expert systems, case-based reasoning systems, machine learning systems, Bayesian classifiers, quantitative models, statistical models, and decision trees—the most frequent being expert systems and ML systems), the variety of applications (including classifications of gestures, images, text, decision making, program debugging, music recommendations, financial accounting, strategy games, command training, robotic agents, non-player character agents, patient diagnosis), various hypotheses concerning the relation of explanation to fundamental cognitive processes, links to learning, users of explanations, limitations and foibles of explanatory reasoning, individual differences in explanatory reasoning (83–96, 71–73, 75–77, 102). Not only is there an issue about the different ways in which the performance of an application in AI might be evaluated (69), there is also the fact that it is far from obvious what explanations would mean to an individual, and whether they might prioritize current justifications or future understandings of the system (67).

Adadi and Berrada (2018) provide a vast survey of XAI, referring to the commercial, ethical, and regulatory reasons why explanations are needed; that is, explain to justify, to control, to improve, and to discover. They also discuss the explainability methods in terms of local vs. global, intrinsic vs. *post-hoc*, model-specific vs. model-agnostic. See especially the table summarizing the key concepts of XAI (52141). An XAI word cloud that they provide (52140) has Interpretable ML and Explainable AI as the most prominent terms in the literature. Hoffman et al. (2018) review the literature on explainable AI from various disciplines (though not economics) referring to work that sees explanation as a continuous process, as a co-adaptive process, in terms of triggers, as self-explanation, as exploration, and as contrast cases. They also refer to the evaluations of explanations in AI by goodness criteria, by use of mental models, and by performance attributable to the explaining process *via* controlled experimentation.

Arrieta et al. (2019) provide a comprehensive overview of XAI concepts, taxonomies, opportunities, and challenges. Especially useful are the following overviews: an overall tabulated picture of the classification of machine learning models in relation to their level of explainability (90), the taxonomy of reviewed literature and trends relating explainability techniques to different machine learning models (93), and a schematic presentation of XAI in deep learning (99). They note that interpretability of black box ML is important to ensure impartiality in decision-making, robustness to potentially adversarial perturbations, and as an insurance that only meaningful variables infer the output (83). They point out that the literature asks for a unified concept of explainability and combining connectionist and symbolic paradigms would be a way to address this challenge, however there exist conflicts of goals and the need for collaborative sense-making. While terms such as understandability, comprehensibility, interpretability, explainability, transparency are used in related ways in the literature on AI and XAI, they suggest that understandability is the most essential concept in XAI (84–85).

The differing approaches to explainability are further accompanied by a skepticism about their usefulness; for instance, whether too much transparency can lead to information overloads, whether visualizations can lead to over-trust or mis-readings, whether ML systems can actually provide natural language rationales in any real sense, and that different people need different explanations anyway (Heaven, 2020). The competing demands and performance-accuracy trade-offs relevant for the provision of explanations are also considered in the Royal Society policy briefing (2019) but with the caution that explainability alone cannot answer questions about accountability (since convincing but misleading explanations might create misplaced trust), leading to the question whether unintelligible systems should be deployed at all in certain areas (The Royal Society, 2019, p. 21–23).

Additional skepticism about the cost of explanations is emphasized in Doshi-Velez et al. (2017) who refer to the role of explanation for accountability in law and the trade-off between the usefulness and the cost of explanations. Beginning from the colloquial sense of any clarifying information as potentially being an explanation, they simply see explanation as “a set of abstracted reasons or justifications for a particular outcome, not a description of the decision-making process in general” (4). For them, explanation generation is a question of system design and they suggest that explanation systems should be considered distinct from AI systems (16–17), in order to create opportunities for industries that specialize in explanation systems in human-interpretable terms without this affecting the accuracy of the original predictor.

There are debates among philosophers over the epistemological interpretation of explanation in AI. For instance, Paez (2009) seeks to restore the factivity condition (the explanans and explanadum must both be true) and sets out the desiderata for belief revision model for explanation which should be based on a realistic notion of a belief state, quarantine inconsistencies in a belief state by solving the problem locally, not be tied to a single notion of explanation and have enough formal resources to express causal, functional, intentional, and probabilistic statements, as well as be able to distinguish between individual facts and general laws. Paez (2019) argues that the search for explainable AI decision-making must be reformulated as part of a broader project of offering an understanding in AI. On the other hand, O'Hara (2020) argues that explanation should be seen as a process and Walton (2008) suggests that explanation should be understood as argumentation in a dialogue model. Fumagalli and Ferrario (2019) put forward a teleosemantic account of explanation, such that content is determined by a success condition. Miller (2020) has argued for a structural-model approach to contrastive explanation.

Another focal area is where to place the human in XAI. Kirsch (2017, p. 1) drives this point home by asking “explain to whom?”. She argues that “comprehensibility and explainability must always be regarded in the context of a specific use case… adequacy can only be determined by interaction with users”. Mueller et al. (2020) overview of the principles of explanation and human-AI systems also focuses on “the need for use-inspired human-focused guidelines for XAI” (1), underscoring the importance of human-centered design that can anticipate the expectations of explanation (when and what to explain) in a responsive manner while maintaining a role for critique in the process of system self-explanations to develop understanding. The work in psychology on cognition and biases uses the evidence from human reasoning to argue for their input in interpretable models of complex AI systems. As an example, Byrne (2019, p. 6280) suggests that experimental research on the role of counterfactuals in human comprehension can be used for “selective navigation through the natural ‘fault-lines’ can ensure that an agent provides analyses that resonate with those that people produce”.

As the foregoing demonstrates, literature on XAI has been overwhelmingly positivist in the ways in which it has made connexions to social sciences. This might be expected given that most researchers within AI have a background in computing, mathematics, information sciences, natural sciences, and perhaps in psychology or philosophy. As we know from sociology of scientific knowledge (SSK), the nature of the knowledge produced by a field is influenced substantially by who the practitioners within the field are, and what the field takes as given. Adadi and Berrada (2018, p. 52142) note that “rarely in literature we come across the term ‘social science’… Yet explanation is a form of social interaction and clearly, it has psychological, cognitive and philosophical projections. Based on the conducted analysis, ideas from social science and human behavior are not sufficiently visible in this field”. A notable exception is Miller (2019) who provides a detailed overview of insights from the social sciences (but not economics) on the nature and structure of explanations, with a four-fold finding that explanations are contrastive; they are selected from among many; reference to probabilities does not matter as much as the reference to causes; and explanations are social.

The messiness of organizing meaningful interaction of AI with the human interface in human–AI systems lies precisely in the difficulties posed by the “social” that precedes the science in social sciences. Humans are ultimately “systems” only in the boundary defying sense of superlatively complex systems, and not in any strict sense of mechanistic systems. Unlike the natural sciences where patterns, regularities, laws and such-like may obtain in observable, quantifiable, and replicable ways, in the social realm, human behavior internally and within and across collectives in different settings is neither straightforwardly observable, measurable, nor even predictable across different populations. It is further subject to dynamic manipulation and change *via* various means of representation, approximating roughly what is termed “constructivism”. Social sciences proper, beyond psychology and philosophy, have grappled with various ways of coming to terms with creating narratives that purport to “explain” diverse outcomes to themselves and others.

No work on explanation in AI so far, including that on social sciences, has indicated any cognizance of, or contained any dialogue with, the idea of explanation or its epistemological underpinnings in economics. This is remarkable because these disciplines have overlapping histories and many commonalities, plus there is a peculiar problem of explanation in relation to theories, models, and the real-world in economics too. In the next sections, I address this.

## Economics, Economists, and Explanation

Economists, unless they are aware of the history, methodology, and philosophy of the discipline, see the “explanations” provided by their economic theories, or more precisely models, as the form that the theory takes, as self-evident. But, for most people who are affected by the explanations provided by these theories, this idea of explanation seems counterintuitive to say the least. Only a small number of economists have puzzled about the curious nature of “explanation” in their field.

Practitioners within the field see “theory” as the constructing of a hypothetico-deductive mathematical model about any economic process as it exists in the real world but with a variety of caveats (such as holding other things constant or ceteris paribus) and assumptions about human behavior that may defy empirical plausibility and realism. The economic method is one of rational choice theory, cost-benefit analysis, game theory, modeling human behavior as an exercise in utility-maximization. The model ends up “explaining” how the outcome that was the subject of theorizing was the result of rational actors behaving under specific assumed conditions. In other words, the rational maximizing behavior of economic agents as modeled would inevitably result in the outcome. Any social outcome, from discrimination to unequal division of labor and more (see Kaul, 2007, p. 170–172) can in this way be shown to be the result of rational behavior on the part of individuals.

The contrast between economics and other social sciences in terms of how the field consists of elaborate deductive systems and formal logical-mathematical methods was noted by Papandreou (1959) who concluded that economists construct models, not theories, and that these models are unable to be refuted by empirical data, and that these models therefore are “*strictly explanatory*” (Papandreou, 1959, p. 1099, emphasis original)^2^. He asks, “Of what use are these constructions if they do not provide us with quantitative information?” (1097). Although meant to be universal, when contradicted, the comeback for the model (and this idea of explanation) is that it was not meant to explain that instance. In his view, economic models can only explain, and only in the instances where they are confirmed, but they cannot predict; nonetheless, economists are willing and often called upon to make predictions so that predictions made on the basis of models are therefore susceptible to subjective orderings. Contrary to finding such a state of affairs as unsatisfactory, Papandreou (himself a Berkeley economist and later an important Greek politician) suggested that the use of such models that provide this particular kind of explanation can work as long as we give to empirical content “a liberal interpretation”.

Another eminent economist, Kornai (1971), had argued that since economics is not a logical and mathematical discipline, but rather a real science, its fundamental task is “the *explanation* of a critically important aspect of economic *reality*” (7, emphases original). Another noted welfare economist, Kaldor (of the Kaldor-Hicks compensation criterion among other things) penned a strong critique of “equilibrium economics” (a field of Walrasian provenance and most clearly identified with the Nobel economist Debreu), arguing specifically that equilibrium theory a la Debreu did not have any explanatory power or relevance in relation to actual economic outcomes. Kaldor (1972, p. 1237, emphases original) was of the view that although Debreu described his subject matter as “an *explanation* of the price of commodities resulting from the interaction of the agents of private ownership company”, by the term “explanation” he means “a set of theorems that are *logically* deducible from precisely formulated assumptions”. The problem that this kind of “pure theory” poses is that it does not intend to describe reality, yet “these increasingly abstract and unreal theoretical constructions are also increasingly taken on trust” (1239). Kaldor concluded that growth in the twentieth century was the result of active government intervention and not the sorts of explanations provided by equilibrium theory. The larger and continued problem today is the legitimization of self-regulating mechanistic systems as the appropriate way of structuring and governing economies.

I have identified these examples to show how prominent and *practicing* economists who worked with mainstream economic theory (and not just economists who concern themselves with questions of economic methodology or philosophy of economics) have long puzzled about the nature of theorizing, the purpose of models, and ultimately the status of explanation within economics. The discipline has a very peculiar and rather unsatisfactory account of explanation in relation to its core workaday practice of doing economic theory, and yet this kind of explanation must be understood to be “good explanation” in order for the field to function with a degree of legitimacy and public trust in the nature of the expertise.

In addition to the examples of well-known economists, there are several other economists who have explicitly alluded to the nature of explanation within the discipline at different times^3^: for instance, Brennan (1979, p. 920) to attempt reconciliations between opposing sides on the role of fact-value or positive-normative divides “to recognize the necessity of teleology in explanation but the inability of pure empiricism to determine human purposes”; Puu (1969) to distinguish and elaborate upon the nature of teleological and causal explanations in specific areas of economics; Guala and Salanti (2001) to discuss the reliance upon the rational choice model even by experimental economists; Kincaid (2012) to provide a discussion of issues surrounding explanation in different sub-fields of economics; Johnson (1996) to consider deductive vs. inductive reasoning in neoclassical and institutional economics; Marchionni (2017) to offer a categorization of the problems in model-based explanations and discuss their relationship to the method or to the nature of the field; Jackson (2002) to provide a qualified defense of functional explanation in economics. Lawson (1997, 2008) bears special mention for his advocacy of the use of contrastive explanations (later contrast explanations) since the social world is an open system where modus ponens/tollens (inferences, if A, then B to affirm or deny) regularities are not properly ascertainable.

Both teleology and the notion of causality have been a central part of the concerns that lie behind the discussions of explanation in economics. Economists have long recognized that the nature of the discipline involves the social world, however they have also always aspired to be producing *scientific* theories. These scientific theories in the field have taken the form of the hypothetico-deductive model that cannot be falsified. The relationship of this model to empirical reality is not mediated by the input of data but by the introduction of assumptions. The *representative rational economic man* of this model is not the human individual but is rather an abstraction imbued with the properties not dissimilar to the AI agent (in terms of rationality, information processing, constant move toward optimization and equilibria). The support for the positive-normative divide (“economics is a positive science about what is, not what ought to be”) is accompanied by a reliance upon teleological explanations, while at the same time being aware of the ideological evaluations that such explanations can import into theorizing. There is a clear awareness of the intellectual satisfaction of properly causal explanations, yet a superbly convoluted understanding of “explanation” that emerged over time. Drawing upon Kaul (2007), I now provide an original genealogy of this notion of explanation by spotlighting certain important epistemic shifts and consequent methodological compromises in the history of economic thought.

## Genealogy of Explanation in Economics

In Conan Doyle's *A Case of Identity*, Watson queries Holmes on how he was able to read a good deal in someone, which was quite invisible to him. Holmes replies, “Not invisible but unnoticed.” In a similar vein, my endeavor is to read the development of explanation in economics in a way that picks up the links that are not invisible, but unnoticed. The accumulated epistemological heritage of what we see as economics testifies to its tension-ridden theoretical and scientific status. The conventionally understood aim of economic theories is to provide explanations, but the issue is what do we mean by an explanation in economics? This is an important question if we need to analyze why and how a particular notion of explanation came to be center-stage in economics. If we genealogically excavate the concept of explanation as I do here, we find that its mutations and inflections indicate the changing ways in which what counts as knowledge and the terms of access to it have been understood.

John R. Hicks^4^, in his unconventionally titled book *Causality In Economics* (1979, p. 1), wrote: “Causality and economics, which I have joined in my title, are words that are not often found together. Causality, the relation between cause and effect, is thought to be the business of philosophers; economists, though they often talk about effects and sometimes (perhaps less frequently) about causes, are usually content to leave the question of the meanings of these terms to others.” Hicks went on to distinguish between what he called “Old Causality” and “New Causality.” At the time of the early origins of modern economics in the Enlightenment era, the notion of causality underwent a change from being associated with responsibility to being associated with explanation. Whereas, previously causes were thought of as actions by an agent, human or not, this theological-legal notion of causality was later transformed into causality becoming a matter of explanation, free from the purview of praise or condemnation.

Hicks explained this as follows: “Causation in terms of the New Causality could only be asserted, if we have some theory, or generalization, into which observed events can be fitted; to suppose that we have theories into which *all* events can be fitted, is to make a large claim indeed. It was nevertheless the claim that thinkers of the eighteenth century, dazzled by the prestige of Newtonian mechanics, were tempted to make…a complete system of natural law seemed just round the corner” (Hicks, 1979, p. 8–9, emphasis original). What Hicks calls New Causality can be seen as a product of the enlightenment epistemology—with its faith in the complete system of natural laws composing an invisible order to the machinelike universe, the belief in the value of generalizations and comprehensibility of knowledge. To assert causation was no longer to imply the responsibility in terms of a human or supernatural agent, it was simply an application of theory, an exercise in explanation.

The notion of explanation underwent a process of change from its Aristotelian origins into a Newtonian (also called Galilean) format that was aspired for by the classical tradition of economists up until Mill. Adam Smith contrasted the two traditions thus: “in Natural Philosophy or any other Science of the Sort we may either like Aristotle go over the Different branches in the order they happen to cast up to us, giving a principle commonly a new one for every phenomenon; or in the manner of Sir Isaac Newton we may *lay down certain principles known or proved in the beginning from whence we account for several Phenomena connecting all together by the same Chain*.—This latter we may call the Newtonian method is undoubtedly the most Philosophical, and in every science whether in Moralls or Natural Philosophy etc., is vastly more ingenious and for that reason more engaging than the other” (in Coleman, 1995, p. 137, emphasis added). Smith favored the Newtonian method or explanation by general principles. I agree with Hicks in how even the title of Smith's book *An Inquiry into The Nature and Causes of the Wealth of Nations* is indicative of thinking in terms of the New Causality; Economics had cast its lot by committing to the search for generalizations or “laws” as the basis for asserting something about the causes of events. However, the appeal of Aristotelian explanations was not so easily done away with. The legacy of teleological explanations continues to play its part.

The distinction between the Aristotelian and the Newtonian/Galilean tradition hinges on the contrast made by characterizing the Aristotelian tradition as having a view of scientific explanation which is teleogical and finalistic, so that it focuses on human efforts to “make facts teleologically or finalistically understandable”, while the Galilean tradition conceives of scientific explanation as causal and mechanistic in its attempts to focus on human efforts to “explain and predict phenomena” (Von Wright, 1971, p. 2–3). Positivism^5^ is related to Galilean tradition and von Wright (Von Wright, 1971, p. 4) identifies its features in terms of: methodological monism or the belief in the unity of method amidst the diversity of the subject matter of scientific investigation; exact natural sciences especially mathematical physics as the standard or ideal of perfection to which all knowledge must aspire; a view of scientific explanation which is in some sense “causal” so that it places an emphasis on explaining individual instances by subsuming them under hypothetically assumed more general laws of nature, including human nature, and a characteristic attitude toward “finalistic explanations” or Aristotelian explanations (Von Wright defines these as “attempts to account for facts in terms of intentions, goals, purposes”) that involves either rejecting them as unscientific or attempting to purge them of their “‘animist’ or ‘vitalist’ remains” and transforming them into causal explanations.

What is the Aristotelian framework? Referring to Ruben (1990, p. 77–109 and 110–154), we can understand the general Aristeotelian theory of explanation as based on a metaphysical notion of substance with four important correlates—element, structure, motion originator, and goal. The requirements of scientific explanation are different from those of general explanation because scientific knowledge involves knowing both the explanation of a thing and the necessity of that knowledge—that is, knowledge of the bare fact as well as the reasoned fact. Scientific explanation is deductivist, so that it is a demonstration of a deductively valid syllogism from necessary premises to a necessary conclusion, and must in addition obey six stated conditions, one of which requires that the premises must be more familiar than the conclusions in nature and to the knower (i.e., a move from familiar to the unfamiliar).

This leads to a trilemma of explanation (and of epistemic justification) so that explanation must either infinitely regress, or be circular, or ultimately self-explanatory or inexplicable. The ultimate self-explanatory first principles of a science can be seen to be the result of a process of induction from particulars. The object of science is not to explain the particulars, but to explain as necessary the general laws of which the particulars may be an instance. Here, a *tension* between the general and the particular is visible in the context of scientific knowledge, viz., the particular instance in some way contributes to the first principles of explaining general laws in scientific knowledge, but it is the general laws, which explain the particular cases. Also, because the first principles of science are arrived at by a process of induction, they cannot be a priori, and so are not necessarily self-evident.

Economics (including classical political economy), uncomfortably saddles the intersection of practical and productive knowledge with (aspirations to be) scientific knowledge. Historically, the spheres of operation of these two kinds of knowledge were seen as separate and separable, but the enlightenment emphasis on “scientific” as a criterion for all knowledge, and the successes of natural sciences, set in motion a vigorous intellectual ferment to align the “social” with the natural sciences. Such an alignment, although never completely effected, nevertheless was repeatedly attempted.

Mathematical formalism in the late nineteenth and then twentieth centuries, although neither necessary nor inevitable (and always much resisted, see Mirowski, 1991), provided great service in giving a successful appearance of such an alignment, especially in economics. Being a deductivist system, mathematics (and associated mathematical models) could provide an intellectually convenient bridge in the uneasy crossing of the general/particular divide in economics by reinforcing the Aristotelian idea of explanation of general laws, rather than particular instances, as being the objects of scientific knowledge. Except that unlike Aristotelian first principles of science (which are composed of non-demonstrable understanding, are self-explanatory but are *not* a priori), the first principles of economic science are often curiously a priori.

Aristotelian (or finalistic) explanation ultimately relied upon non-demonstrable understanding, or self-explanatory (but not a priori) first principles of science, which could perhaps be had from particulars by induction, but could not be explained without the general laws. The positivist attitude toward this idea of explanation was an empiricist alternative, so that explanation could either be rejected as an unscientific concept, or, reconstructed in line with empiricist principles. The physicist Duhem, for instance, rejected explanation as a metaphysical idea, which transcends experience and could result in subordinating physical theory to metaphysics: “The aim of physical theory is merely to summarize and classify logically a group of experimental laws ‘without claiming to explain these laws’…‘a physical theory is not an explanation. It is a system of mathematical propositions, deduced from a small number of principles, which aim to represent as simply, as completely, and as exactly as possible a set of experimental laws” (Duhem, 1977, p. 19, in Ruben, 1990, p. 113). In this view, explanation is connected with a non-empirical conception of reality, it has no place in science; the system of mathematical propositions deduced from a small number of principles represents—a set of experimental laws.

Smith rejected the Aristotelian concept of explanation as I mentioned before, but in the antecedents of modern economics until the late nineteenth-century, there was a desire to adopt the Newtonian/Galilean version aligned to positivism, even as Aristotelian or teleological notions of explanation persisted. It was not until JS Mill in the nineteenth century that this tension between the positivist aspirations of the discipline and the appeal of Aristotelian frameworks was explicitly addressed. Mill (unlike the route to reject explanation chosen by Duhem) reconstructed explanation in line with empiricist principles.

Mill, who is a significant precursor of modern economics, recast explanation into a format which was adopted by twentieth century positivists, and by the neoclassical tradition of economists. Mill was part of an empiricist tradition (Hobbes, Bacon, Locke, Berkeley, Hume), which sought to criticize or reformulate concepts or ideas which could not be traced directly to experience. For Mill, explanation did not reveal deeper mysteries of nature; he did not want to explain in any final sense. Explanations, according to him, needed laws, which he saw as uniformities, either simultaneous or successive. If the uniformities of successive phenomena are to be causal, then they are invariable and unconditional regularities of experience. Mill rejected any non-empirical idea of causation as metaphysical and also differentiated between the ordinary and scientific meanings of explanation; while ordinary explanation replaced the unfamiliar by the familiar, scientific explanation replaced the familiar by the unfamiliar. Ruben (1990, p. 115) comments that Mill often talks about “events and facts as what explain and are explained”, noting that *facts* did not figure in Plato's or Aristotle's ontology of explanation. What is one to make of the notion of a “fact”?

Mill introduced the notion of the “fact” in relation to explanation. Mill's usage of the notion of a “fact” is both symptomatic and constructive of a larger role that the epistemological unit of a fact has played in modernity. Poovey (1998) sees the category of the factual in most modern sciences in the West as positioned between the phenomenal world and systematic knowledge, as a result of which, “the epistemological unit of the fact has registered the tension between the richness and variety embodied in concrete phenomena and the uniform, rule-governed order of humanly contrived systems”. Mill's elevation of deduction over induction in the emergent social sciences owed to a recognition of the *tension between observed particulars and theoretical or systematic knowledge as a problem that required a professional (or disciplinary) solution* (Poovey, 1998, p. 3, 317–325).

At the beginning of the period roughly understood as the enlightenment, Bacon was important in elevating the observed particular, from which one could move to making generalizations which constituted systematic knowledge. But induction had its problems, and one can see Hume's philosophical formulation of the problem of induction in the 1740s as a belated effect of Bacon's empiricism. Poovey (Poovey, 1998, p. 14) points out that although Hume himself did not see this problem as particularly troubling, his formulation of this tension between the observed particulars and systematic knowledge allowed the peculiarity written into the modern fact to be conceptualized as such. In the nineteenth century, Mill (and others such as McCulloch and Herschel) were important in the formulation of a disciplinary solution to this problem, it was a solution which involved, “turning the task of knowledge production in the rapidly professionalizing sciences over to so-called experts” (3).

These experts eventually introduced the formulation to gradually elevate “rule-governed, autonomous models over observed particulars” (Poovey, 1998). This reformulation occurred at different moments in different disciplines, but as a result it was ensured that (Poovey, 1998, p. 3, emphasis original): “After the late nineteenth century, at least in the natural and social sciences, expert knowledge producers sought not to generate knowledge that was simultaneously true to nature and systematic but to *model* the *range of the normal* or sometimes simply to create the most sophisticated models from available data, often using mathematical formulas”. The methodological compromise involved creating taxonomies of knowledge, classes of experts, usage of statistics and mathematical modeling as an effective solution. This is something that is otherwise just usually stated but not genealogically excavated^6^.

Mill was an influential figure in effecting the methodological compromise following on from tensions in enlightenment epistemology. Mill saw deductive inference as circular and as founded upon some sort of non-deductive inference, so that deduction cannot advance knowledge, nonetheless, instead of relating explanation to induction, he epistemically downgraded explanation from its non-empiricist pretensions (Ruben, 1990, p. 130–1, 137). Mill does not see explanation as being able to answer the why question, we can never ever really know, all that we can do is to fit facts into wider patterns by deductive arguments. This is exactly the state of the discussion in contemporary economics, which does not really care much about what one does when one explains, all that one can do as a good scientist is to fit patterns by deductive mathematical models.

Finally, Mill also originates the “symmetry thesis”, the idea that explanation and prediction are symmetrical so that they are both identical in content of their product, which is a deduction. This is another lasting influence of Mill on economic thought. Debates in economic methodology have long discussed this concept (see Blaug, 1980a,b; Rosenberg, 1992). Mill's cementing this particular kind of deductivist basis for explanation paves the way for abstractions such as nature, society etcetera to be incorporated into models of a pared down notion of knowledge which does not aim to represent knowledge as being both true to nature and systematic, but as a deductivist modeling of abstractions. This deductivist modeling of abstractions as a basis for “theoretical” knowledge in economics then changes the very notion of theory and transmogrifies it into what is ubiquitous as the contemporary model.

The genealogy of explanation that I have provided here highlights the Aristotelian ideas inflected through the enlightenment, and nineteenth century methodological compromises effected as resolution to tensions in enlightenment epistemology. The result was a positivist social science with theoretical understanding constituted by modeling abstractions in a deductivist mathematical format. This then required the fixing of individual choice as the focus of analysis, and characterization of individual choice in a way as to make it mathematically tractable and commensurable. It further required these abstractions to function in a credible way, a task for which the role of metaphors is crucial.

Now, refer back to the puzzling reflections of practicing economists and occasional philosophers^7^ about the nature of economics, and their dissatisfaction with the relationship between the model and the real world, and the inability to provide proper explanations in spite of the claims to being a social science. The account that I have provided here answers those paradoxes by tracing how we came to adopt this particular notion of explanation in terms of the tensions between the general and the particular that it reconciles and the methodological compromises that it enables between deductive scientific claims and empiricist requirements. This genealogy of explanation constellates a trajectory of intellectual thought that is unfamiliar to those in AI who look to the social sciences for an understanding of explanation. The longer historical reference in XAI only goes as far back as Hempel, and it has been my intention here to excavate beyond the standard Hempelian backdrop^8^.

Consider also that for Friedman (1953), a Chicago School Nobelist and preeminent and dogmatic monetarist, judging the success of economic theory by its comparison to reality was unpalatable because realism is unattainable. Even Samuelson (MIT economist, Nobelist, author of arguably the most famous textbook on economics) felt compelled to disagree with Friedman on the “realism of assumptions” issue^9^, whereby Friedman held that the assumptions of economic theorizing, and therefore of model, did not need to be realistic so long as they predictively successful (so that people, households, firms could be behaving “as if” it were the case).

Thus, basing the theory as an effort to model the range of the normal relies upon the typical individual economic agent with certain (a priorily) assumed characteristics. Empirical validation can be sought in the epistemological strength emanating from the law of large numbers. The role of statistics in generating the realm of the average or the normal was important in the late nineteenth century. “Whereas British philosophers since Hume had asked how one could reason from observed particulars to final causes or from observed particulars to general laws, *after statistics began to be equated with the law of large numbers*, philosophers as well as ordinary readers began to ask how one could conceptualize free will, given that the *regularities that emerge from ‘numerical calculations'* seemed to leave so little room for volition, for morality, or for ethics of any kind” (Poovey, 1998, p. 325, emphasis added).

The epistemological history of economics as a social science has insights to offer because it can help us to comprehend *why* an explanation is understood to be what it is conceived of as being, and also that it does not necessarily need to inevitably be so. I argue that critical and genealogical accounts of *how the idea of explanation itself has shifted in meaning* over time can be useful as a signpost. Explanation does not mean understanding, and this is true not just in the usual sense that explanations provided might not be understood by the users of the AI interface if they are not experts but are rather individuals impacted in different ways by the decisions made by the machine. It is also true in the deeper sense that the understanding of what is meant by an explanation, for instance in economics, can also be shown to have shifted over time in coherent ways. These *systematic changes in consensual understanding of what occurs when something is said to have been “explained”*, have reflected the methodological compromises that were rendered necessary to serve different perspectives in the discipline over time. This is a salutary reminder that the search for XAI is not exhausted by what explanation is or what explanation should be, but can include the epistemological ground of what explanation *can be*.

The longue durée view of how the idea of explanation shifted in economics can help with understanding the methodological compromises that can be manifest as tensions between the salience of the general/particular, or to put it in AI terms, the global/local. It can also help with appreciating the rise of the position of the “expert”; this expert was a human one in the late nineteenth century, but is increasingly likely to be AI in the twenty-first century.

## Economics and AI Reconsidered

Economics as a social science has had a complex and contested history of its development as a field of knowledge. In lay understanding, economics and business are often conflated; economics, or more precisely, economic theory refers to mainstream economics, principally the neoclassical school of thought, with its origins in the marginalist revolution, which occurred toward the end of the nineteenth century (see Kaul, 2007, p. 32–33). It was at the start of the twentieth century that the change of terminology from political economy to economics came about and the discipline was reconstituted with a conscious intent to create a study of society or econom-ics in the way that physics was the study of the natural world (Perelman, 1996). Neoclassical economics, as proto-energetics, borrowed its metaphors from mid-nineteenth-century energetics (see Mirowski, 1989, p. 224 for term-for-term translations between mechanics and economics where “energy” in physics becomes “utility” in economics, see also Schabas, 2005).

Why think of Economics and AI together? Let me count the reasons. To begin with, there is the historical perspective on the intimate links between the development of economics and of machine learning from the middle of the twentieth century onwards. I bring together the two separate accounts, by Mirowski (2002) and Mendon-Plasek (2021), which highlight these rare histories for neoclassical economics and machine learning, respectively. In Cold War/post-war U.S, the research agendas of mechanistic conceptualization and realization of human behaviors, motivations, and interactions was furthered in both these disciplines from similar institutions, funding bodies (for instance RAND) and even individuals in mathematics, game theory, engineering, and decision sciences who moved between the disciplines.

In tracing the story of how economics became a cyborg science, Mirowski notes “Machine rationality and machine regularities are the constants in the history of neoclassical economics; it is only the innards of the machine that changed from time to time” (Mirowski, 2002, p. 9–10). The footprint of a mechanistic and computational view of describing and predicting human behavior became clearer with the development of “neoclassical price theory, game theory, rational expectations theory, theories of institutions mechanism design, the nascent program of ‘bounded rationality,’ computational economics, ‘artificial economies,’ ‘autonomous agents,’ and experimental economics” (Mirowski, 2002, p. 9). Meanwhile, there was the impulse toward pattern recognition in order to mechanically identify significance and reproduce human judgment based upon the beliefs that “there was little difference between looking for patterns in the natural world and looking for patterns in human society” (Mendon-Plasek, 2021, p. 33).

Beyond methodology, the important epistemological question here in these commonalities and overlaps is the way in which the object of enquiry and the nature of explanation was simultaneously understood in these disciplines, including by their own practitioners. Each of these disciplines, as they evolved, gained policy traction from their systematization of messy realities, however these methodological compromises necessitated a peculiar link between the empirical—whether as data for ML/AI or abstract assumed characteristics of a rational economic man for economics—and the scope of generalizations that were held out as a promise. It is not surprising at all, therefore, that Parkes and Wellman (2015, p. 268) write “without offering any judgment on the question of how well rationality theories capture essential human behavior, we note the irony in the prospect that social science theories may turn out to apply with greater fidelity to non-human agent behavior”. The specific reference they make is to economics and how the perfectly rational agent of neoclassical economics is sought to be constructed by AI researchers under the guise of a synthetic *homo economicus*.

It is worth remembering the continuity in the fundamental worldview of present computational/complexity economics and the antecedent versions of neoclassical economic theory that go back to the late nineteenth century, and an even longer fetish for the “machine ideal”. It is with the era of what is called European enlightenment and the onset of modernity that machines came to be associated with the “exercise of reason in a knowable world” (Kaul, 2007, p. 34). In this era, machines were objects in the way that they had always been, but they also became exceptionally powerful metaphors for schematizing comprehension. From this time onwards, it was possible to assert that the world was ultimately knowable if approached by proper scientific methods; moreover, the complexities of entities such as “nature” or “society” would be rendered understandable by using the metaphor of the machine. The close correspondence between the notion of a “system” and that of a machine meant that viewing something as a system became tied to comprehending the mechanistic nature of systems. Prominent philosopher-economists such as Adam Smith maintained that “systems in many respects resemble machines. A machine is a little system…A system is an imaginary machine” (in Coleman, 1995, p. 132).

There are at least two immediately relevant implications of viewing social processes as mechanistic systems: First, machines are a whole more than the sum of their parts, they produce ultimate benefits that could not have been produced had the parts not been set up in a particular way. When we use this analogy to view systems in society as imaginary machines, the hierarchies of social order in this imaginary machine become just “different” systemic levels that serve a purpose in the larger scheme of things. Thus, when we view “the economic system”, we are locked into certain conceptions of human beings, material interactions, and ultimate benefits that cannot easily be challenged and are best not interfered with.

Unsurprisingly then, Smith's laissez-faire or free market doctrine judges a system best which is least regulated, so that the “invisible hand” guides the marketplace as each individual pursues their self-interest. Economists themselves still continue to see themselves in varying relations to engineering (see Mariotti, 2021, p. 555 for three different paradigms suggesting the evolution of economics and engineering nexus; see also Klein, 2020). In her lecture titled “Economist as plumber”, Duflo (2017) draws upon her fellow economist Bannerjee's summary of the nature of the field where he writes of how economists tend to think of the world in “machine mode” by making assumptions about the non/running of the machine and wanting to find out the button that will get the machine started rather than stepping into the machine to figure out the adjustments.

The notion of machines can be used to serve the ideals of “efficiency” or “rationalization” of systems. While the efficiency and rationalization in systems can be understood in ways that are neither innate nor universal (such as by asking “efficient for whom”, see Barker, 1995), the use of the machine metaphors dispense with these concerns since mechanistic systems came to be understood as self-evidently superior. What had started at the dawn of the Enlightenment era as “the Cartesian view of life as mechanism” had, by the late nineteenth century, developed into a fully-fledged romance with the machine as the ideal (Connor, 2002). Daston and Galison (1992) designate the emergence of this ideal as the moralization of objectivity and the mechanization of science. The notion of what it meant to be objective was now distinctively mechanical in its methods, indicative of self-restraint in its morals, and individualized in its metaphysics (Daston and Galison, 1992, p. 84).

Machines came to be celebrated as the paragon of human virtues, since they had in abundance what humans lacked such as “freedom from will, accuracy and eternal vigilance” (Daston and Galison, 1992; Kaul, 2007, p. 77–81). Today, we see this rationale at work in reference to “clear benefits to algorithmic decision-making; unlike people, machines do not become tired or bored…and they can take into account orders of magnitude more factors than people can” (Mehrabi et al., 2021, p. 1151). What machines did was seen to operate in a vacuum of meaning; in spite of this cultural fantasy, automation has always been gendered, raced, and served dominant economic interests, both then and now. In parallel, the rise of statistics indicated the important epistemological role played by the idea of numbers in the aggregate, a precursor to today's big data.

We are still confronting the many ways in which this understanding of what factuality is, or to put it in Poovey's terms, “the modern fact” that presents a particular problem of how numbers are approached as being pre-interpretive and yet also non-interpretive (1998). The impact of statistics upon of economics over time was not simply in the greater availability of data, but also in the ways in which the historical rise of statistics shaped the link between “countability” and the idea of “general and stable truths about societies and the individuals that compose them” (Nirenberg and Nirenberg, 2021, p. 217).

It is instructive to remember that in nineteenth century economics, there was an intense debate on “rigor” and “practicality” as rival ideals of quantification (see Porter, 1994, p. 128–172 on this). Many different political economists, most notably Ricardo, were targeted as being too theoretical and lacking in rigor. Their critics advocated for recourse to statistical factuality and induction as well as mathematical exposition in order to guard against the errors of reasoning and theoretical excesses. On the other hand, the economists inspired by engineers and physicists were interested in quantifying economic magnitudes in practical terms by drawing upon scientific vocabularies of the effectiveness of engines. Thus, “a system of economic practice that will permit scientists to judge the productivity of machines and labor, as well as to improve them. In this economics, statistics of factories, workers, and production meant something. Quantification could aid administration, could guide the improving activities of engineers and reformers…” (Porter, 1994, p. 142–143).

The economics of mathematized utility, or Walrasian general equilibrium (on Walras's economics and its other pioneers, see Mirowski and Cook, 1990, cf. Samuelson, 1952) combined the disdain for theoretical excess with a reverence for mathematical analysis, but with a focus on formal mathematical models rather than inductive statistical work. The result of this was a structuring of expert understanding about human social processes that concerned work, saving spending, production, consumption and so on (i.e., those deemed to be of relevance to economic understanding), in the form of abstract formal mathematical models that are disconnected from practical everyday experience or data and further make conclusions that are conditional upon several assumptions required for the model to work.

This particular way of constructing knowledge about economic behavior was enabled by the work of machine metaphor, which lends an understanding of the economy as a mechanistic system made up of rational self-interested utility maximizing individuals simplified within the language of mathematics, while erasing their lived histories, reinforcing the structural and systematic discriminations they face, and making invisible the power differentials that are core to the ways in which they can exercise their ostensibly rational choices.

If researchers in AI ask “explain for whom?” when arguing for the need to keep the human in the loop, critical economists have also asked, “whom or what does the representative individual represent?” (Kirman, 1992). The idea of the human individual is made up of, to quote Nirenberg and Nirenberg (2021, p. 220), “every new potential ‘brick’ of sameness” with which “new claims of certainty and determinism rise in our ways of thinking about the human and the world”. If today, we do not have adequate ways of explaining the results of the staggering developments along specific methodological/technological lines in these disciplines back to the individuals themselves, then we might think back to figures like von Neumann and Morgenstern who “set up to treat humans and their desires like mathematical objects: transitive, apathic, quantifiable” (Nirenberg and Nirenberg, 2021, p. 228).

Mirowski quotes the computer scientist Joseph Weitzenbaum's statement that “the avatars of artificial intelligence (AI) tend to describe a very small part of what it means to be human being and say that this is the whole” (2002, p. 3). This lack of holism it is also particularly evident in the standard assumption of ceteris paribus (other things being equal) in economics that is neither realistic, nor can the predictions be easily mapped onto the real world. The issue of the symmetry thesis (familiar from Mill), the idea that explanations and predictions have the same logical structure, therefore poses a problem for both disciplines. Moreover, the predictions have less than definite meaning because of the restrictive conditions or specific reasoning they are obtained with, from AI systems that learn correlations but cannot predict based on the right reasons (for an example of link between hotel occupancy and prices, see Chipman, 2018) to economic models that seek to explain any social outcome as a result of rational choice by individuals and claim only to be uncovering causal regularities by doing so, may end up concluding that discrimination could benefit those who are discriminated against by forcing them to accumulate more human capital (see Bergmann, 2002, p. 65–66; Kaul, 2007, p.130–31).

Partly as a result of the ways in which economics and AI have developed with multiple internal fragmentations, there is a shared quest for proper disciplinary identity. It is often said that “economics is what economists do”, and likewise “AI is what AI researchers do”. A microeconomist wishing to avoid attempting to define economic theory, stated “perhaps it's like pornography, in that you know it when you see it” (Allen, in Kaul, 2007, p. 6). In a similar manner, Wang (2008) points out how AI is marked by disagreements not only about the best solutions to problems but also on what the problem is, noting that AI is simply what AI researchers do is a valid descriptive definition, but not a working one. Wang (2008) writes that there are various ways in which the meaning of intelligence in AI can be defined: by structure, by behavior, by capability, by function, or by principle; each of these working definitions corresponds to a different level of description and a certain level of abstraction, and each has different implications in terms of research goals, contributions, domains and pathways.

The problems of “internal fragmentation” and “external recognition” (sometimes termed the “AI effect”) that Wang outlines, are also palpable in economics (see Kaul, 2007, passim). Equally, the ethical concerns raised by the knowledges legitimized by both these fields resonate with public everywhere. In the case of economics, the public concerns about how economic theory legitimizes an unequal status quo are widely known. An overview of citizen juries and public dialogues on machine learning and AI also indicated that “people's views on particular applications of machine learning were often affected by their perception of who was developing the technology, and who would benefit” (The Royal Society, 2019, p. 20).

In the twenty-first-century, further changes have led to the development of complexity economics (see Holt et al., 2011), which shares many, but not all, of the underlying neoclassical assumptions (see Arthur, 2021, p. 143 for a comparison of the differences between neoclassical and complexity economics). Mirowski (2002) had already signposted the ways in which a machine theory of mathematics reoriented the practice of economics, noting, two decades ago, the possibility that “markets as automata are coming to work station near you” (545).

Agent-based computational economics and a complexity economics framework sees the economy in terms of process and seeks to capture the feedback between micro and macro structures by modeling markets made up of diverse non-human agents who can simulate phenomena to allow strategies to evolve in order to study networks, change transmission, systemic risks, norm revolution, and policy gaming (see Arthur, 2021, p. 136–142, Van de Gevel and Noussair, 2012; Gogas and Papadimitriou, 2021, p. 64–73). But, Mariotti (2021, p. 561–2) refers to the challenges posed by algorithmic collusions (where AI autonomously learns to adopt collusive pricing rules) and points out the problems ahead for prospects of the economies of AI in terms of market manipulations *via* personalized dynamic pricing, granular forms of indirect price discrimination, and digital doubles of individuals. These are at base ethical and political questions that cannot be simply technologically solved.

Moreover, the positivist conceptualization of the fact-value distinction is at work in both these disciplines (and is also often challenged by practitioners). Post-positivist philosophical traditions in science have alerted us to the theory-ladenness of observations, the under-determination of theory by empirical data, and the role played by values in the selection of research questions, the constitution of research communities, the standards of epistemic justification, and the significance of individual experiences. Feminist epistemological interventions are therefore an important resource for both of these disciplines (see Kaul, 2007; Johnson, n.d.) to be able to come to terms with the ways in which ostensibly neutral constructions such as models and algorithms are value laden.

Doshi-Velez et al. (2017, p.18) state that “AIs have perfect memory and do not suffer from cognitive biases and social pressure”. Even so, it is usual for most researchers to recognize how biases can play a role in AI because they are trained upon human data, that there ought to be debiasing strategies and initiatives for more responsible AI, and there might be conflicting notions of fairness (see Ras et al., 2018; Zhang and Bareinboim, 2018; Fernández and Fernández, 2019, p. 22, Kirchner and Larrus, 2019, p. 5; The Royal Society, 2019, p. 10; Kantarci, 2021; Mehrabi et al., 2021). Fazelpour and Danks (2021) also substantively explain how the use of predictive algorithms can preserve or even compound existing injustices, and “fairness through unawareness” almost never succeeds so that “algorithmic bias is not a purely mathematical problem” and requires engagement with “the messy complexities of the real world”.

The fundamental way in which biases are part of the very design of algorithms is rarely dwelt upon and this point is powerfully made by Johnson (n.d.). “Aspects of these algorithms all the way down to the very design decisions that produce them are suffused with normative implications, and thus, questions of their production, use, and evaluation belong properly within the purview of ethical theory”; algorithms are both useful and value laden (Johnson, n.d.: 27–28). Machines may lack qualia, but algorithms are not value free even in their material design.

## Conclusion

Garfinkel (1981) asked, “If social science is the answer, what is the question?” Analogously, we could ask: if AI is the answer, what are the questions? AI, in its many variations^10^, will increasingly shape the geographies of our knowledge about ourselves and the world, make sense of our experience as we interface with digital and real entities, decide what we can access, and curate what we can, and do, care for. The prior hierarchies and vulnerabilities that already signify our episteme, risk becoming further datum for the systematically variable outcomes that will be installed by the logics of the machine. In other words, “the insight and intelligence with which we address machine learning systems today will be the linchpin of future bad laws that we must later protest” (Mendon-Plasek, 2021, p. 57–58). The assessment of technologies is a complex matter, and as Sclove (1995, p. 4–5) points out, this is not simply reducible to the questions that are typically asked by newspapers, public-interest groups, corporate leaders, and governmental bodies—i.e., Is it workable? What are the economic costs and benefits? How are they distributed? What are the associated risks? Are there implications for national security? Beyond all these doubtlessly important questions, there is a fundamental overall question that is crucial in guiding technological change: “What would be the impact on our desired form of society if individuals, or the community, were to adopt one set of technologies rather than another?”; the conclusion is not that we must rid ourselves of technology altogether, but that there must be “possibility of eliciting alternative technologies more compatible with the kind of society or communities in which people wish to live” (Sclove, 1995, p. 6–7).

Mumford (1964, p. 2, 5) presented the coexisting natures of two technologies—the authoritarian technics (being system-centered, immensely powerful, but inherently unstable) and the democratic technics (being human centered, relatively weak, but resourceful and durable). The authoritarian technics in the modern era, he argued, were restored at the same time as the regimes of absolute governments were overthrown so that military coercions were reproduced in the organizations of factories and with scientific ideologies that liberated them from theological restrictions or humanistic purposes.

“Through mechanization, automation, cybernetic direction, this authoritarian technics has at last successfully overcome its most serious weakness: its original dependence upon resistant, sometimes actively disobedient servo-mechanisms, still human enough to harbor purposes that do not always coincide with those of the system” (Mumford, 1964, p. 5). He referred to the lack of visible personality as the center of authority [in a manner reminiscent of the philosopher of totalitarianism, Arendt (1969), who referred to the “rule by nobody”]. The success of human surrender to authoritarian technics is paradoxically owed to the fact that “if one surrenders one's life at source, authoritarian technics will give back as much of it as can be mechanically graded, quantitatively multiplied, collectively manipulated and magnified” (Mumford, 1964, p. 6).

Technical systems are persuasively interwoven with the conditions of modern politics and technical arrangements are forms of order (Winner, 1980); the histories of architecture, city planning, and public works testify to this. Technologies are inherently political, “the things we call ‘technologies’ are ways of building order in our world” (Winner, 1980, p. 127). A particular point of historical relevance to AI is the range of choices that have to do with “specific features in the design or arrangement of the technical system after the decision to go ahead with it has already been made” (Winner, 1980, p. 127). The link between technologies and politics is important, and yet far from straightforward, especially if we consider that people are often “willing to make drastic changes in the way they live to accord with technological innovation at the same time they would resist similar kinds of changes justified on political grounds” (135). This is resonant with the contemporary present where AI is altering the landscape of our lives.

The ascendancy of economics as the science of society was historically achieved by defining the quotidian economic experience through its scientific construal even when it failed the tests of realism or ethics. The historical genealogy of the discipline mirrors many of the present dilemmas. Examples here include the contestations of rival ideals such as “rigor” and “practicality” for the field; the different levels of knowledge and disciplinary purpose between those who worked with data as opposed to those who “do theory”; the rise of experts who became progressively professionalized and unintelligible to most; a gradual delinking from context and history in favor of uniformity and abstraction; and an obfuscation of the questions of power in favor of technocratic scientisms that were seen as being “factual”. The beauty of the method and the aesthetics of the conclusions relied upon the creation of an imagined abstract human being as the norm; the rational, maximizing, atomistic individual agent of the kind that is not alien to AI.

The problem of explanation in AI, like the problem of explanation in economics, is not merely a technical one; perhaps not only, or really, a problem of satisfactory explanation provision. It is interwoven with questions of competing epistemological and ethical choices and related to the ways in which we choose sociotechnical arrangements and offer consent to be governed by them. Historically, a mix of various factors—including developments in computing technologies, perceived strategic need for massive investments in such areas, seduction of data as helpful in responding to essentially political questions in a technocratic manner, and the longstanding appeal of machine ideals as being superior to human beings (since machines do not tire, they require fewer resources, they don't ask questions, they are stupendously efficient at pattern recognition and so on)—combined to create the innovative AI present that we are in.

Questions of knowledge are also at the same time questions of social order (Shapin and Schaffer, 1989) and knowledge creation is always already a form of ethics, politics, strategy, subjectivity, reason, and being (Kaul, 2007). A combination of strategic political and commercial economic rationales over time have thus combined to result in the neural politics of pareidolia that we are evermore being accustomed to, and this is ultimately what needs explanation, or at least better colligation. In this last, storytelling about the changing nature of what counts, or can count, as an explanation, and the intertwining of economics and AI rationales is crucial. This present article is a modest step in that direction.

## Data Availability Statement

The original contributions presented in the study are included in the article/supplementary material, further inquiries can be directed to the corresponding author.

## Author Contributions

The author confirms being the sole contributor of this work and has approved it for publication.

## Conflict of Interest

The author declares that the research was conducted in the absence of any commercial or financial relationships that could be construed as a potential conflict of interest.

## Publisher's Note

All claims expressed in this article are solely those of the authors and do not necessarily represent those of their affiliated organizations, or those of the publisher, the editors and the reviewers. Any product that may be evaluated in this article, or claim that may be made by its manufacturer, is not guaranteed or endorsed by the publisher.

## Footnotes

^1^Traditional AI methods and tools (“logical predicates, ontologies, artificial neuronal networks, ant algorithms, and others”) are based on formalized approaches and use denotative semantics; this ‘weak AI' is contrasted to ‘strong AI’ that relies on cognitive semantics (Raikov, 2021, p. 117–118). Strong AI, or AGI (artificial general intelligence) refers to AI that “emulates human-level reasoning and competence” (Pearl, 2019: n.p.). For important original debates concerning machines, brains, and programs, see Searle (1980).

^2^The discussion here pertains to economics, or more precisely, mainstream economics, but not to econometrics where modeling proceeds in an augmented form so that “their models… do *not* enjoy the insulation from the impact of data which is characteristic of the basic models of the general economist” (Papandreou, 1959: 1099, emphasis original).

^3^I refer to work where the notion of explanation and why it is necessary or what form it might take or the puzzles that it presents us with, has been explicitly referenced with salience in relation to economics. Notwithstanding titles, I exclude references to work which makes a cursory or tenuous link to economics (for instance Child, 2020). See Kaul (2007) and Nachane (2003) for further references and broader discussions.

^4^Hicks was jointly (with Kenneth Arrow) awarded the Nobel Prize for Economics in 1972. His major texts include *The Theory of Wages* (1932), *Value and Capital* (1939), and *Capital and Growth* (1965).

^5^The alternative view to positivist empiricist enlightenment epistemology is a view characterized as anti-positivism, one strand of which is the hermeneutic tradition. Linked to this is the contrast drawn by the German historian-philosopher Johann Gustave Droysen (1858) between “Eklaren” or explanation and “Verstehen” or understanding (see Von Wright, 1971, p. 5, 172).

^6^For instance, Von Wright (1971, p. 7) states that the “application of mathematical methods to political economy and other forms of social study was an inheritance of the eighteenth-century Enlightenment which found favor with the nineteenth-century positivists”.

^7^For example, the philosopher Reiss (2012) identifies the explanation paradox in economics by the three mutually inconsistent hypotheses: that economic models are false; are nevertheless explanatory; and only true accounts explain. He concludes that the paradox is genuine and likely unresolvable.

^8^According to Hempel, basic scientific explanation is of two types—(a) the deductive nomological (DN) type of explanation, and the later added (b) the probabilistic statistical or the inductive statistical type (IS) of explanation. They are both ideal types in being idealizations/models or rational reconstructions by the standard of which actual explanations can be judged, and are found wanting in some or other aspect. Hempel does not aim to provide a plausible account of how scientists actually explain, rather, the purpose is “to indicate in reasonably precise terms a logical structure and the rationale of various ways in which empirical science answers explanation-seeking- why-questions” (Hempel, 1965, p. 412). See also Kaul (2007, p. 70–73).

^9^For a compelling critique of Friedman on explanation and the nature of economic theory, relevant to the nature of my arguments here, see McLachlan and Swales (1978).

^10^Kotseruba and Tsotsos (2020) review 40 years of cognitive architectures of general AI, a field with an aim to create programs that “could reason across different domains, develop insights, adapt to new situations and reflect upon themselves” (21) providing a taxonomy (Emergent subdivided into connectionist logic systems and neuronal modeling; Hybrid subdivided into fully integrated and symbolic sub-processing; and Symbolic) of 84 such architectures based on representation and processing. These architectures aim to represent AI's epistemology. The newest paradigm: ML methods, and especially data-driven deep learning, has had huge practical success in several domains, but such techniques are “not widely incorporated into existing cognitive architectures” (68). On the other hand, a smaller set of cognitive architectures explicitly pursue the goal of artificial general intelligence or AGI (22), for example the fully integrated NARS (see Wang et al., 2018).
